# Cocaine inhibition of nicotinic acetylcholine receptors influences dopamine release

**DOI:** 10.3389/fnsyn.2014.00019

**Published:** 2014-09-04

**Authors:** Alexandra Acevedo-Rodriguez, Lifen Zhang, Fuwen Zhou, Suzhen Gong, Howard Gu, Mariella De Biasi, Fu-Ming Zhou, John A. Dani

**Affiliations:** ^1^Department of Neuroscience, Baylor College of MedicineHouston, TX, USA; ^2^Department of Pharmacology, University of Tennessee College of MedicineMemphis, TN, USA; ^3^Department of Pharmacology, Ohio State UniversityColumbus, OH, USA; ^4^Departments of Psychiatry, Perelman School of Medicine, University of PennsylvaniaPhiladelphia, PA, USA; ^5^Department of Neuroscience, Perelman School of Medicine, University of PennsylvaniaPhiladelphia, PA, USA

**Keywords:** substantia nigra, ventral tegmental area, addiction, mesolimbic, voltammetry, nAChRs

## Abstract

Nicotinic acetylcholine receptors (nAChRs) potently regulate dopamine (DA) release in the striatum and alter cocaine's ability to reinforce behaviors. Since cocaine is a weak nAChR inhibitor, we hypothesized that cocaine may alter DA release by inhibiting the nAChRs in DA terminals in the striatum and thus contribute to cocaine's reinforcing properties primarily associated with the inhibition of DA transporters. We found that biologically relevant concentrations of cocaine can mildly inhibit nAChR-mediated currents in midbrain DA neurons and consequently alter DA release in the dorsal and ventral striatum. At very high concentrations, cocaine also inhibits voltage-gated Na channels in DA neurons. Furthermore, our results show that partial inhibition of nAChRs by cocaine reduces evoked DA release. This diminution of DA release via nAChR inhibition more strongly influences release evoked at low or tonic stimulation frequencies than at higher (phasic) stimulation frequencies, particularly in the dorsolateral striatum. This cocaine-induced shift favoring phasic DA release may contribute to the enhanced saliency and motivational value of cocaine-associated memories and behaviors.

## Introduction

Midbrain dopamine (DA) projections to the striatum comprise an important neuronal system mediating the initiation of drug addiction (Bonci et al., 2003; Wise, 2004; Hyman et al., 2006). The most well known action of the addictive drug cocaine is its inhibition of monoamine transporters, such as the DA transporter (DAT), with an affinity of about 500 nM (Ritz et al., 1987; Pristupa et al., 1994; Jones et al., 1995). In the brains of abusers, however, cocaine often reaches concentrations of 5–10 μM for considerable durations, and higher concentrations are achieved for shorter times depending on the route of administration and other factors (Mittleman and Wetli, 1984; Evans et al., 1996; Ward et al., 1997; Fowler et al., 1998). In addition, cocaine inhibits α4β 2-containing (α4β 2^*^) nicotinic acetylcholine receptors (nAChRs) with an IC_50_ in the range of 5–15 μM (Damaj et al., 1999; Francis et al., 2000). This inhibition may be significant because α4β 2^*^ nAChRs (often in combination with α6) are highly expressed in DA neuron somata and terminals (Mansvelder and McGehee, 2000; Jones et al., 2001; Champtiaux et al., 2003; Wooltorton et al., 2003; Quik and McIntosh, 2006; Zanetti et al., 2006), and nAChRs have been shown to regulate the frequency dependence of DA release (Zhou et al., 2001; Rice and Cragg, 2004; Zhang and Sulzer, 2004; Exley and Cragg, 2008; Zhang et al., 2009a). Intertwined with the DA neurons and their axons, cholinergic neurons in the midbrain and brainstem project to the substantia nigra and ventral tegmental area (VTA) (Woolf and Butcher, 1986; Gould et al., 1989; Oakman et al., 1995), while cholinergic interneurons in the striatum innervate locally (Woolf and Butcher, 1981; Zhou et al., 2001; Nelson et al., 2014), providing the endogenous neurotransmitter ACh to the nAChRs in these DA areas.

In the striatum, DA release is normally dependent on both afferent spikes along DA fibers and nAChR activity at axonal and presynaptic locations (Zhou et al., 2001; Grady et al., 2002; Salminen et al., 2004; Zhang et al., 2009a). The nAChRs normally increase the initial DA release probability, and regulate the frequency dependence of DA release (Rice and Cragg, 2004; Zhang and Sulzer, 2004; Exley and Cragg, 2008; Zhang et al., 2009a). Cholinergic interneuron activity may directly facilitate DA release from DA axon terminals in the dorsal and ventral striatum (Cachope et al., 2012; Threlfell et al., 2012). Inactivation of nAChRs on the DA fibers decreases the DA release probability and increases the phasic to tonic DA ratio (Rice and Cragg, 2004; Exley and Cragg, 2008; Zhang et al., 2009a). Based on those published findings, we hypothesized that cocaine acts via nAChRs to regulate DA signals beyond the expected inhibition of DATs.

## Materials and methods

### Mice

Male and female C57BL/6J mice from The Jackson Laboratory (Bar Harbor, Maine), β 2-subunit knockout (KO) mice (Xu et al., 1999), DAT knockin mice having DATs that are insensitive to cocaine (Chen et al., 2006), and their wild-type (W-T) littermates were used in our present study. The mutant mice were generated, maintained, euthanized and genotyped according to established procedures (Xu et al., 1999; Chen et al., 2006) and in accordance with national and institutional guidelines. Experiments on the mutant mice and their W-T littermates were performed double blind.

### Fast-scan cyclic voltammetry (FCV) in striatal brain slices

**Figure 1:**
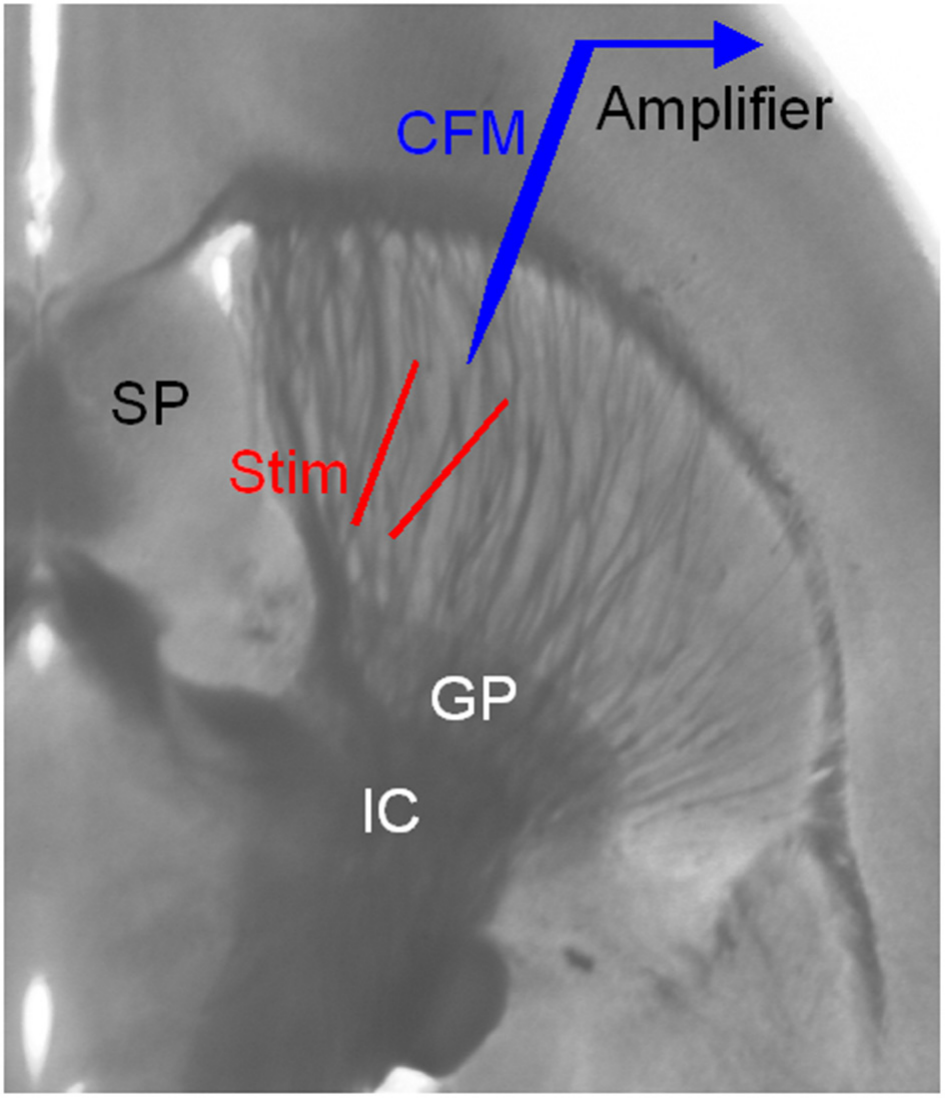
**Arrangement for electrical stimulation and FCV recording in striatal brain slices**. The striatum is easily identified by its anatomical location and the distinct fiber bundles in horizontal brain slices. Local electrical stimulation in the striatum was delivered using a bipolar tungsten electrode. The two tips of the stimulating electrode were ~150 μm away from each other and from the carbon fiber microelectrode (CFM) tip. GP, globus pallidus; IC, internal capsule; SP, septum.

For these studies, horizontal brain slices (Figure 1) containing the striatum (400 μm in thickness) from mice 1–3 months old were cut on a Leica VT1000 or Leica VT1200s vibratome (Zhang et al., 2009a). Anesthesia, handling, and experimental procedures followed our established techniques at 30°C (Zhou et al., 2001).

FCV was performed with home-made carbon-fiber electrodes constructed from P55S carbon fibers of 10 μm diameter (Amoco Polymers, Greenville, SC). Axopatch 200B amplifier, pClamp 9 software, and Digidata 1320A interface (Axon Instruments) were used to acquire and analyze data. The holding potential was 0 mV between scans. Scans of 20 ms duration were applied at 10 or 20 Hz. The scans were from 0 mV to −400 to 1000 to −400 to 0 at a rate of 300 mV/ms and were sampled at 50 kHz. The peaks of the voltammograms were plotted over time and converted to concentrations by post-experiment calibration with 0.1–5 μM DA standards.

Local electrical stimulation in the striatum was delivered using a bipolar tungsten electrode (Figure 1). The two tips of the stimulating electrode were ~150 μm away from each other. The tip of the carbon-fiber recording electrode was 100–200 μm away from the two tips of the stimulating electrode. The stimuli were relatively weak (0.1–0.2 mA for 0.1 ms) helping to keep the recording stable and minimize local interactions. The interval between single pulses was usually 100 s. Additional types of stimulation were used: tonic stimulation of 4 pulses at 4 Hz, phasic stimulation of 4 pulses at 20 Hz, and paired pulses separated by 50 ms. Cocaine was bath-applied for 10 min and then was washed out.

### Patch clamp recording of midbrain DA neurons

Coronal or horizontal midbrain slices, 200–300 μm in thickness and containing the substantia nigra pars compacta (SNc) and/or the VTA, were prepared from 15 to 30 day old mice according to established procedures (Pidoplichko et al., 1997). Visualized recordings were made at 30°C using an Axopatch 200 and pClamp data acquisition and analysis software.

nAChR-mediated currents in putative DA neurons were induced by pressure application of 1 mM ACh using a Picospritzer (General Valve) attached to a puffer pipette (Pidoplichko and Dani, 2005). The puffer was ~30 μm from the neuron while ACh was applied (25 psi for 100 ms). Then, the puffer was retracted 100–200 μm between ACh applications by a computer-controlled manipulator to prevent leak-induced desensitization (Wooltorton et al., 2003; Pidoplichko and Dani, 2005). For this experiment, the brain slices were bathed in a normal extracellular solution containing (in mM): 125 NaCl, 2.5 KCl, 1.25 NaH_2_PO_4_, 25 NaHCO_3_, 2.5 CaCl_2_, 1.3 MgCl_2_, and 10 D-glucose that were continuously bubbled with 95% O_2_ and 5% CO_2_. The intracellular solution contained (in mM): 135 KCl, 0.5 EGTA, 10 HEPES, 2 Mg-ATP, 0.2 Na-GTP, and 4 Na_2_-phosphocreatine. pH 7.25, 280–290 mOsm. To record the fast voltage-activated sodium current (I_Na_), the presumed nigral DA neuron (based on its slow firing rate at ~1 Hz in cell-attached mode, see Ding et al., 2011) was held at −90 mV and then stepped to 0 mV for 10 ms. For this experiment, 2.5 mM CaCl_2_ were substituted by 2.5 mM MgCl_2_, and extracellular NaCl was reduced to 25 mM, 20 mM TEA and 5 mM 4-AP were used to inhibit K currents; the following Cs-based intracellular solution was also used that contained (in mM): 135 CsCl, 0.5 EGTA, 10 HEPES, 2 Mg-ATP, 0.2 Na-GTP, 4 Na_2_-phosphocreatine with pH was adjusted to 7.25 with CsOH.

**Figure 2:**
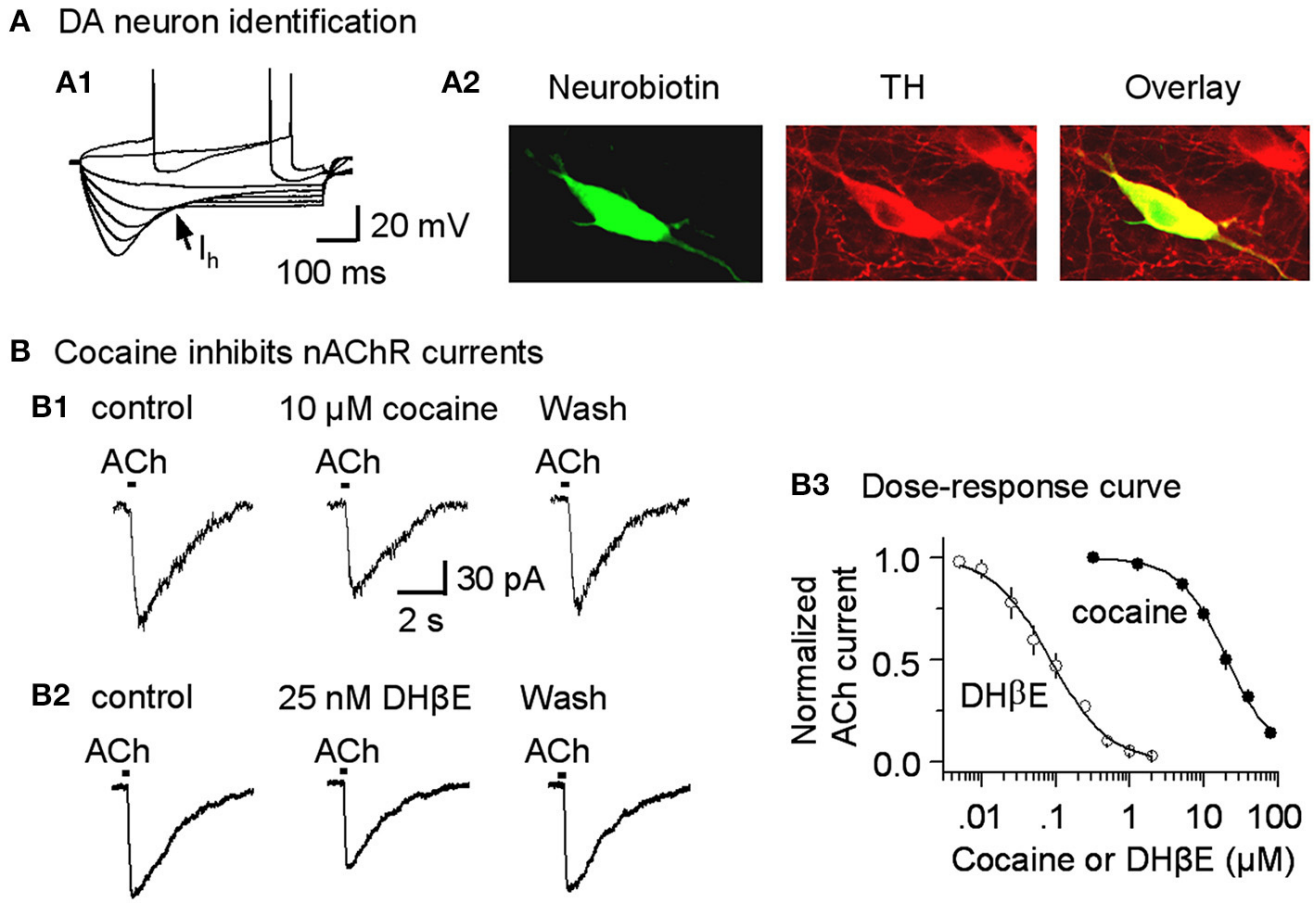
**Cocaine inhibits nicotinic currents in midbrain DA neurons from W-T mice**. **(A,A1)** The membrane properties of presumed DA neurons from the lateral VTA or SNc were typical of DA neurons with a strong I_h_ sag (arrow). Injected current was 40 pA/step. Spikes were partially truncated for display. **(A,A2)** These midbrain DA neurons were backfilled with neurobiotin (green) and labeled for TH (red). The co-incidence of neurobiotin and TH is shown as yellow (Overlay), confirming the DA neuron identification. **(B,B1)** Three traces show nicotinic currents induced in a DA neuron by pressure application of ACh (1 mM, 100 ms pulse) under control conditions (left), with 10 μM cocaine present (middle), or with 25 nM DHβ E present (right), which was applied after recovering from cocaine inhibition. The recordings were made in the presence of 1 μM atropine at a holding potential of −70 mV. **(B,B2)** Examples showing DHβ E inhibits nicotinic currents in DA neurons. **(B,B3)** The dose-response relationships for inhibition of nAChR currents. The curves through the data were produced with IC_50_ = 19.8 μM and Hill coefficient = 1.3 for cocaine, and IC_50_ = 82 nM and Hill coefficient = 1.1 for DHβ E with *n* = 3–9 per data point.

Neurobiotin (0.2%) was included in the recording electrode and allowed to diffuse into the cell using approaches described previously (Pidoplichko et al., 1997). After recording, the brain slices were fixed in 4% paraformaldehyde in 0.1 M phosphate buffer. Double immunostaining was performed according to established procedures (Pidoplichko et al., 1997; Neuhoff et al., 2002). Tyrosine hydroxylase (TH, a marker for midbrain DA neurons) was detected with a sheep anti-TH primary antibody and rhodamine red-X-tagged donkey anti-sheep secondary antibodies (Figure 2A2). Neurobiotin was detected with red Cy2-tagged strepavidin antibody (Figure 2A2). Sections were examined on a Bio-Rad confocal laser-scanning microscope.

### Chemicals

All chemicals including cocaine-HCl were purchased from Sigma Aldrich (St. Louis, MO) or Tocris (Ellisville, MO). Cocaine was also obtained from the NIH/NIDA's Drug Supply Program.

### Statistics

The data are displayed as the mean and the standard error. Paired *t*-test was used to compare measurements before and during various pharmacological treatments. *P* < 0.05 was considered statistically significant. When applicable, the averaged data points in dose-response plots were fitted with the Hill equation to estimate IC_50_ and Hill coefficient. These and other computations were performed using the Origin analysis and plotting program (Northampton, MA).

## Results

### Cocaine directly inhibits nAChRs on DA neurons

Because cocaine inhibits cloned neuronal nAChRs in heterologous expression systems (Damaj et al., 1999; Francis et al., 2000), we tested whether cocaine inhibits nAChRs expressed on DA neurons of the SNc and the lateral VTA (Picciotto et al., 1995; Pidoplichko et al., 1997; Mansvelder and McGehee, 2000; Wooltorton et al., 2003). DA neurons were initially identified by their characteristic membrane properties. The presumed DA neurons within the lateral midbrain fired spontaneously (2.1 ± 0.3 Hz, *n* = 9), displayed prominent I_h_ currents (Figure 2A1) and had relatively long spike durations (2.7 ± 0.3 ms at the base, *n* = 9). These membrane properties were consistent with commonly recognized DA neuron properties from this area (Neuhoff et al., 2002; Ford et al., 2006; Beckstead and Williams, 2007; Zhang et al., 2010; Ding et al., 2011; Li et al., 2011). These electrophysiologically identified DA neurons were further confirmed by back filling with neurobiotin and subsequently staining for TH (Figure 2A2).

In these DA neurons, pressure application of ACh (100 ms pulse of 1 mM ACh, 1 μM atropine was always present) induced currents (Figure 2B1, left trace) that have previously been characterized as predominantly β 2^*^ nAChR currents, and these currents can be inhibited by 1 μM dihydro-β-erythroidine (DHβ E) (Alkondon and Albuquerque, 1993; Wooltorton et al., 2003). In the presence of a cocktail of antagonists to inhibit DA transporters (2 μM GBR12909), D_2_-like autoreceptors (1 μM sulpiride), and muscarinic receptors (1 μM atropine), bath application of 10 μM cocaine inhibited the nAChR currents (Figure 2B1, middle trace). This concentration (10 μM cocaine) is within the range achieved by cocaine abusers and by animals during self-administration experiments (Mittleman and Wetli, 1984; Evans et al., 1996; Ward et al., 1997; Fowler et al., 1998; Nicola and Deadwyler, 2000). The dose-response curve for cocaine inhibition of nAChR currents from SNc DA neurons had an apparent IC_50_ of 19.8 ± 1.5 μM and Hill coefficient of 1.3 ± 0.3 (*n* = 3–9, Figure 2B3), and the results were statistically the same for nAChRs in the VTA (IC_50_ of 19.5 ± 1.8 μM, Hill coefficient of 1.3 ± 0.2). To indicate the effectiveness of cocaine inhibition, the nicotinic antagonist, DHβ E, was characterized with an apparent IC_50_ of 82.3 ± 4.3 nM and Hill coefficient of 1.1 ± 0.4 (*n* = 3–7, Figures 2B2,B3). These results suggest that cocaine directly inhibits nAChRs on DA neurons and, thus, acts as a weak nicotinic antagonist. Because the predominant β 2-containing nAChRs on the cell body and axon terminals appear to be qualitatively similar (Champtiaux et al., 2003; Salminen et al., 2004; Quik and McIntosh, 2006), cocaine's nicotinic antagonism likely also occurs at DA axon terminals. Since the selective DAT inhibitor GBR12909 was also used in this study, we tested the potential effect of GBR12909 on nAChR-mediated currents as a separate control experiment. We found that GBR12909 (5 μM, the highest concentration we used) did not inhibit nAChR currents in 5 DA neurons tested. Since nAChRs facilitate DA release (Zhou et al., 2001; Rice and Cragg, 2004; Zhang and Sulzer, 2004; Zhang et al., 2009a; Threlfell et al., 2012), our result on cocaine inhibition of nAChRs leads to this question: can cocaine also affect DA release at DA axon terminals?

### Cocaine inhibits DA release evoked by a single pulse

Cocaine is known to inhibit DA reuptake with a K_i_ of about 0.5 μM (Ritz et al., 1987). This effect is also well documented in the FCV literature (e.g., Jones et al., 1995) and readily observed under our current experimental conditions (see **Figures 6A1,B1**), but is not the focus of our present study. Our focus here is to determine if cocaine, at concentrations above what is needed to inhibit DATs, can affect DA release, because at the relatively high concentrations attained by abusers, the previous section suggests that cocaine inhibits nAChRs on DA fibers and terminals that normally regulate DA release. Therefore, we reasoned that cocaine at different concentrations alters the DA signal in qualitatively different manners via inhibition of DATs vs. nAChRs.

To examine cocaine's nAChR-mediated influence over DA signals, we electrically stimulated DA release and monitored the DA concentration using FCV in brain slices containing the striatum. The DA signal was evoked every 100 s in the dorsolateral striatum and was commonly stable for >2 h during our recordings (Zhou et al., 2001; Zhang et al., 2009a). Confounding mechanisms were minimized in all of the experiments by using pharmacological treatments. Sulpiride (1 μM) was used to inhibit DA autoinhibition by D_2_-like receptors. SKF83566 (1 μM) was used to inhibit D_1_-like receptors to prevent changes in cholinergic tone that affects DA release (Zhou et al., 2001). GBR12909 (1–5 μM), which has an IC_50_ ~10 nM vs. cocaine's IC_50_ ~0.5 μM, was used to minimize DA reuptake by DATs. Therefore, pretreatment with GBR12909 (1–5 μM) occludes cocaine's effect on DA reuptake by DATs.

**Figure 3:**
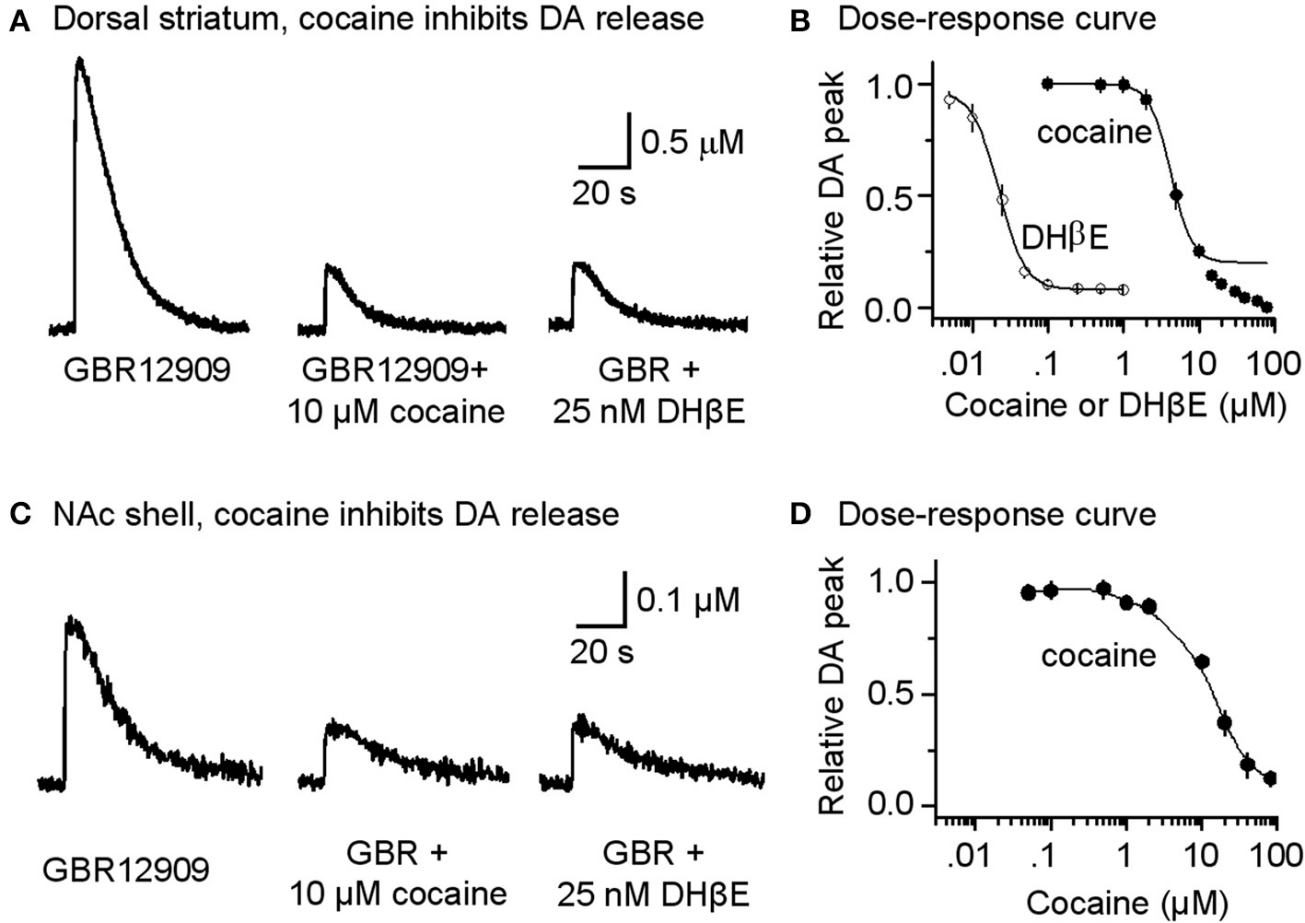
**Cocaine reduces DA release in the striatum from W-T mice**. **(A)** Cocaine inhibits DA release from the dorsolateral striatum evoked by single-pulse (1 p) stimulation measured using fast-scan cyclic voltammetry. The recordings show the prolonged DA signal after DAT inhibition with 5 μM GBR12909 (left trace). DA release was substantially reduced by 10 μM cocaine (middle trace). After recovery from cocaine inhibition, 25 nM DHβ E induced a similar inhibition of the DA signal (right trace). 1 μM sulpiride (D_2_-like antagonist) and SKF83566 (D_1_-like antagonist) were used to block local interactions in the striatum. **(B)** The dose-response relationships for inhibition of DA release. The curves through the data were produced with IC_50_ = 4.3 μM for cocaine and 22 nM for DHβ E both with a Hill coefficient estimated to be 3 (*n* = 4–8 per data point). Data points with cocaine concentrations higher than 15 μM were not included in the fitting because a component arising from a local anesthetic effect was also present. **(C)** Likewise, cocaine inhibits DA release evoked by 1 p stimulation from the NAc shell measured using fast-scan cyclic voltammetry in the presence of 5 μM GBR12909, 1 μM sulpiride, and 1 μM SKF83566. **(D)** The dose-response relationship for inhibition of DA release in the NAc shell.

Under these pharmacological conditions (with DATs inhibited), cocaine at concentrations that would normally inhibit DATs (0.1–1 μM) did not enhance the amplitude or duration of the already prolonged DA signal, as is indicated in the dose-response curve (Figure 3B, filled symbols). In other words, cocaine no longer enhanced the DA signal because DATs were already inhibited by GBR12909. However, at the same concentrations that inhibited nAChRs (Figure 2B1), cocaine (10 μM) and DHβ E (25 nM) substantially inhibited DA release evoked by a single-pulse (1 p) stimulus (Figures 3A,B). Cocaine inhibited the DA signal with an estimated IC_50_ of 4.3 ± 0.3 μM (*n* = 8) and a Hill coefficient of 3 (Figure 3B, filled symbols). The high Hill coefficient is indicative of a cocaine effect on Ca^2+^-triggered vesicular DA release, which has high cooperativity (Lou et al., 2005). Under the same pharmacological conditions, DA release evoked by 1 p also was inhibited by the nicotinic antagonist DHβ E with an apparent IC_50_ of 22.3 ± 1.7 nM and Hill coefficient of 3 (Figure 3B, open symbols). The influence of cocaine over the DA release evoked by 1 p was comparable in the dorsolateral striatum (Figures 3A,B) and in the NAc shell of the ventral striatum (Figures 3C,D).

**Figure 4:**
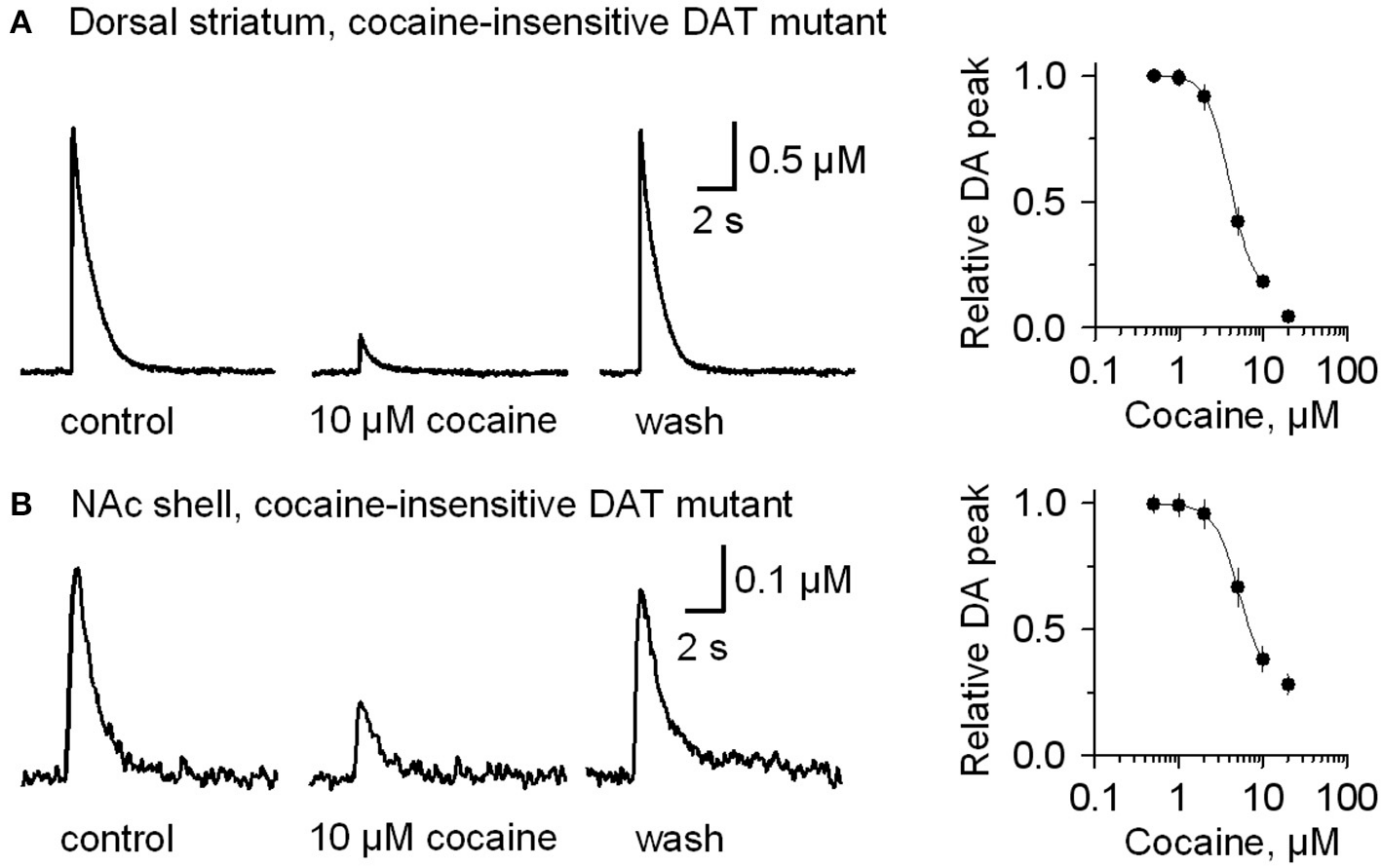
**In mutant mice having cocaine-insensitive DATs, DA release evoked by 1 p stimulation was still inhibited by cocaine (10 μM)**. **(A)** In 10 μM cocaine (middle trace) the DA signal was inhibited when compared to the control (left) or after washout of cocaine (right, wash). The dose-response relationship for cocaine inhibition (extreme right) was fitted with a curve through the data having IC_50_ = 4 μM and Hill coefficient = 3.2. **(B)** Likewise, cocaine (10 μM) inhibits DA release evoked by 1 p stimulation from the NAc shell. The dose-response relationship for inhibition of DA release in the NAc shell by cocaine (extreme right) was fitted by a curve with IC50 = 5.2 μM and the Hill coefficient = 3.0. The traces were collected in the presence of 1 μM sulpiride, and 1 μM SKF83566.

To verify that cocaine was not acting via DAT inhibition when GBR12909 was present in these experiments, we repeated the experiments using mutant mice possessing DATs that are insensitive to cocaine (Chen et al., 2006) instead of using GBR12909. A similar effect was seen when cocaine (10 μM) was applied while stimulating DA release in the presence of 1 μM sulpiride and 1 μM SKF83566 (Figure 4A, dorsolateral striatum; Figure 4B, NAc shell). The DA release to a 1 p stimulation was inhibited by cocaine (10 μM), and the dose-response relationships for cocaine inhibition of DA release is shown to the right in Figure 4. These results verify that cocaine inhibits DA release separately from its influence over DA reuptake or D2-like receptors.

### Cocaine reduces DA release by inhibiting nAChRs

The results to this point show that cocaine can decrease DA release evoked by a 1 p stimulus. Cocaine's ability to inhibit nAChRs suggests that the mechanism of action is via nAChRs on DA fibers or terminals. The nAChRs that normally facilitate DA release contain the β 2 subunit (Zhou et al., 2001; Salminen et al., 2004; Zhang et al., 2009a; Drenan et al., 2010; Exley et al., 2011). In nAChR β 2-subunit KO mice, DA release does not depend on nAChR activation (Zhou et al., 2001; Zhang et al., 2009a). Therefore, if cocaine decreases DA release in response to a 1 p stimulus via nAChR inhibition, then β 2-subunit KO mice should not show this cocaine-mediated effect.

**Figure 5:**
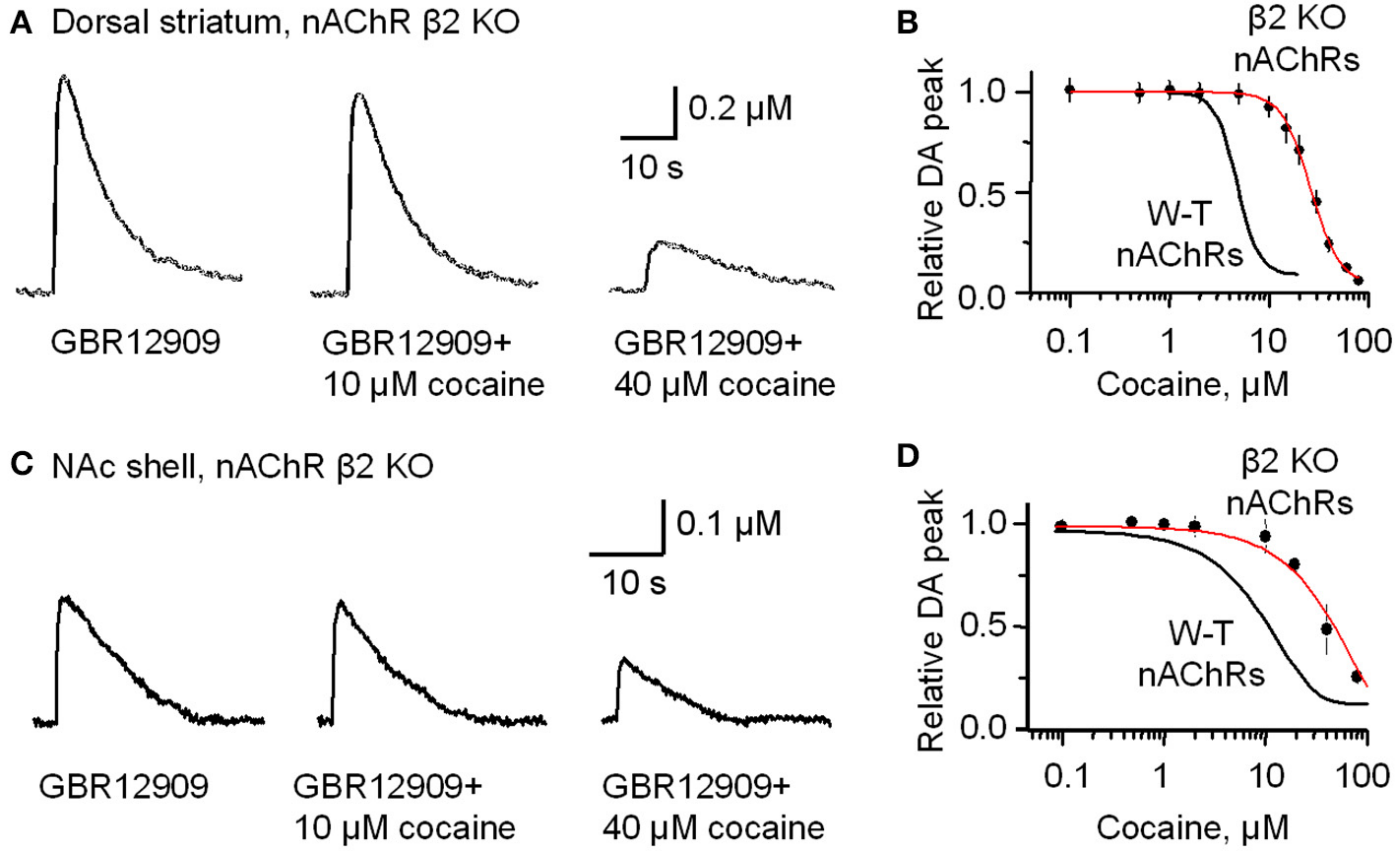
**In β2-subunit KO mice DA release evoked by 1 p stimulation was resistant to inhibition by cocaine (10 μM)**. **(A)** All the slices were bathed in 2 μM GBR12909 to block DATs and to remove complications arising from changes in DA reuptake. The control DA signal (left) evoked by 1 p stimulation shows a prolonged duration caused by inhibiting DA reuptake. In 10 μM cocaine (middle trace) there was slight inhibition of the DA signal. In 40 μM cocaine (right trace) there was a much greater inhibition of the DA signal. 1 μM sulpiride and SKF83566 were used to block local DA receptor influences within the striatum. **(B)** The dose-response relationship for cocaine inhibition of DA release in the dorsolateral striatum of β 2-nAChR KO mice. The curve through the data was produced with IC_50_ = 26 μM and the Hill coefficient = 3 (red curve) (*n* = 4–6 slices). For comparison, the black curve from Figure 1 obtained with W-T mice is given to show the substantial decrease in cocaine-mediated inhibition when β 2^*^ nAChRs were absent. **(C)** Likewise, cocaine (10 μM) did not, but cocaine (40 μM) did, inhibit DA release evoked by 1 p stimulation from the NAc shell again in the presence of 2 μM GBR12909, 1 μM sulpiride, and 1 μM SKF83566. **(D)** The dose-response relationships for inhibition of DA release in the NAc shell by cocaine in β 2-subunit KO mice (red curve) (*n* = 4–5 slices) compared to the W-T mice (black curve).

In the dorsolateral striatum of β 2-subunit KO mice, 1 p stimulation evoked nAChR-independent DA release that had an amplitude 70% smaller than that observed in W-T mice, as has been shown previously (Zhou et al., 2001). The smaller evoke DA signals are observable by comparing Figure 3A (left trace compared to the right trace after inhibition of nAChRs) to Figure 5A (left trace, note the change in scale). In the presence of GBR12909 (1–5 μM) to inhibit DATs, cocaine doses ranging from 50 nM to 5 μM did not affect the amplitude or duration of the evoked DA signal in β 2-subunit KO mice (Figure 5B). A concentration of cocaine (10 μM) that caused a 74.9 ± 7.8% decrease in DA release in W-T mice (Figures 3A,B), produces only 7.6 ± 2.4% inhibition in nAChR β 2 KO mice (Figures 5A,B). In W-T mice, cocaine reduced DA release with an IC_50_ of 4.3 μM (Figure 5B, smooth black curve), but in nAChR β 2 KO mice the IC_50_ shifts to 26.4 ± 2.3 μM with a Hill coefficient of 3.0 (Figure 5B, red curve) (*n* = 4–6 slices for each data points). Comparable results (that may be similarly interpreted) were also obtained in the NAc shell (Figures 5C,D) from nAChR β 2 KO mice.

**Figure 6:**
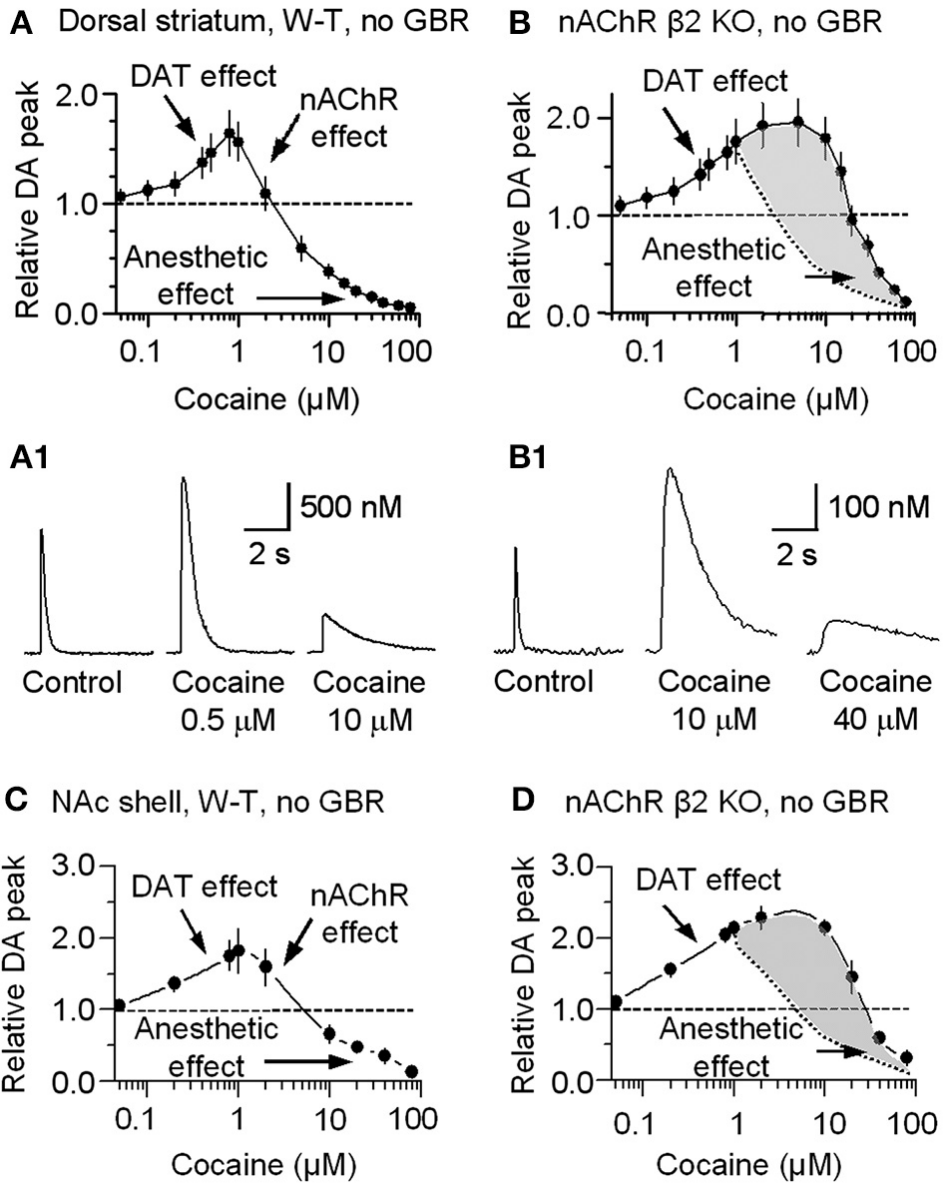
**Concentration-dependent multiple cocaine effects on the DA-release signal evoked by 1 p stimulation in the striatum measured by FCV**. **(A)** In W-T mice DA signals were measured in the dorsolateral striatum while we bath applied cocaine ranging from 50 nM to 80 μM in the absence of GBR12909. At 50 nM to 1 μM, cocaine monotonically increased the amplitude and prolonged the duration of the evoked DA signal, as expected from cocaine's known inhibition of DATs (DAT effect). At cocaine concentrations above 1 μM, the DA peak decreased although the duration was further prolonged. This decrease is hypothesized to arise from cocaine inhibition of nAChRs (nAChR effect). **(B)** In β 2-nAChR knockout (KO) mice in the absence of GBR12909, the DA signal is not dependent on nAChRs. Note that the mid-range cocaine inhibition of the DA amplitude is absent in these measurements (i.e., the nAChR effect is absent). The difference between cocaine's influences in the absence of β 2-nAChRs is shaded in gray with the dotted curve representing the falling phase in **(A)**. **(A1,B1)** Examples of the multiple effects induced by different concentrations of cocaine in WT and in β 2-nAChR KO mice. **(C)** In W-T mice, DA signaling in the NAc shell showed qualitatively similar cocaine effects as seen in the dorsal striatum. **(D)** In β 2-nAChR KO mice, the DA signal is not dependent on nAChRs. The difference between cocaine's influences in the absence of β 2-nAChRs is shaded in gray with the dotted curve representing the falling phase in **(C)**. In **(A–D)**, *n* = 4–7 slices for each data points.

**Figure 7:**
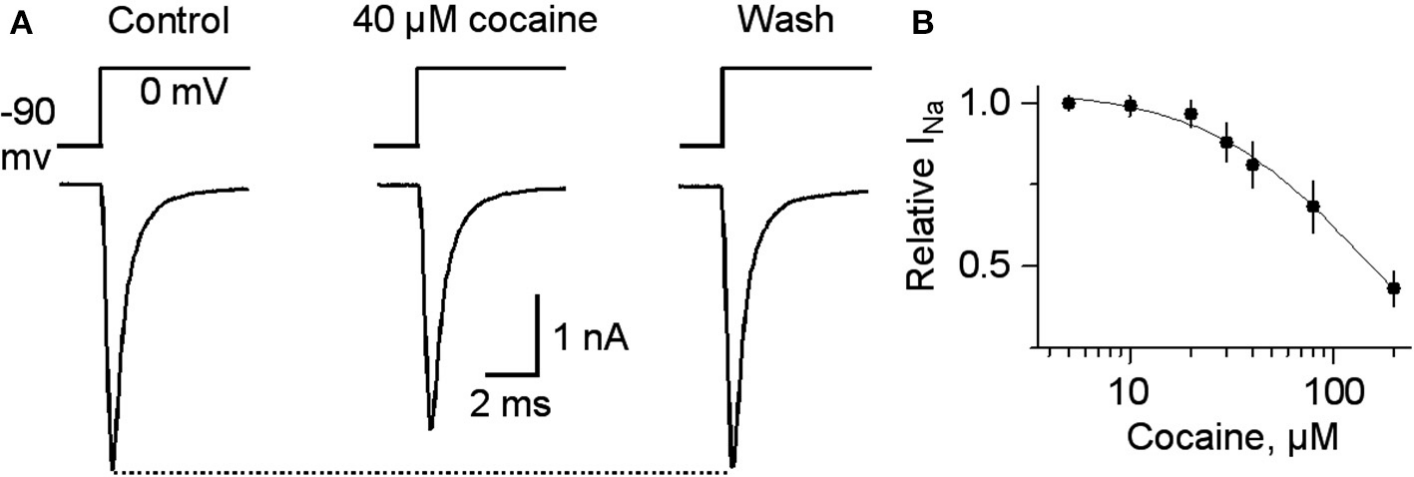
**Cocaine at high concentrations inhibits voltage-gated I_Na_**. **(A)** In 40 μM cocaine, I_Na_ was inhibited compared to the control or after cocaine washout. Holding potential was −90 mV and the testing potential was 0 mV for 10 ms given every 20 s. Extracellular and intracellular solutions are described in the Materials and Methods section. **(B)** The dose-response curve for cocaine inhibition of I_Na_ is shown on the right with a curve through the data with an IC_50_ = 144 and a Hill coefficient of 1.

For completeness, the whole range of cocaine's influence over the 1 p stimulated DA release is shown in the absence of GBR12909 in W-T mice normally expressing nAChRs in the dorsolateral striatum (Figure 6A) or the NAc shell (Figure 6C). At low cocaine concentrations, DATs are inhibited by cocaine, and the DA peak becomes larger (DAT effect, Figures 6A,A1,C). At cocaine concentrations above 1 μM, the DA peak decreases. As indicated by the earlier results, cocaine begins to inhibit nAChRs at this concentration (nAChR effect, Figures 6A,C). When nAChRs are absent (i.e., nAChR β 2 KO mice), cocaine does not begin to inhibit the DA peak amplitude until ≥20 μM (Figures 6B,B1,D). The overall effect of cocaine acting via nAChRs is depicted in Figure 6B. The inhibition of the DA peak by ≥20 μM cocaine likely also involves the local anesthetic effect (Anesthetic effect, Figure 6), arising from cocaine inhibition of voltage-activated channels (e.g., sodium and/or calcium channels) and action potentials (O'Leary and Chahine, 2002). We verified that cocaine inhibited the fast sodium current in DA neuron somata in a dose-dependent manner (Figures 7A,B) that likely contributed to the decreased DA release seen at very high cocaine concentrations (Anesthetic effect, Figure 6). Because DA release depends of the 4th power of the intraterminal calcium concentration that arises from the depolarization caused by voltage-gated ion channels, cocaine's inhibition of voltage-gated channels (e.g., I_Na_ currents in Figure 7) is magnified when observing the effect over DA release (Figure 6).

### Cocaine reduces paired-pulse depression of DA release dependent on nAChRs

The results presented above indicate that cocaine acts as a nAChR antagonist at DA fibers and terminals in the striatum and via this mechanism reduces DA release evoked by a single pulse or low frequency stimulation. This reduction in DA release is, of course, relative to the release that would be seen if cocaine did not slightly inhibit nAChRs. Inhibition of nAChRs also has been shown to reduce paired-pulsed depression of DA release (Rice and Cragg, 2004; Zhang and Sulzer, 2004). Paired-pulse depression largely depends on the initial neurotransmitter release probability. The higher the initial release probability, the stronger the paired-pulse depression (Zucker and Regehr, 2002). Therefore, if cocaine is inhibiting nAChRs, it should decrease the initial DA release probability and reduce paired-pulse depression.

**Figure 8:**
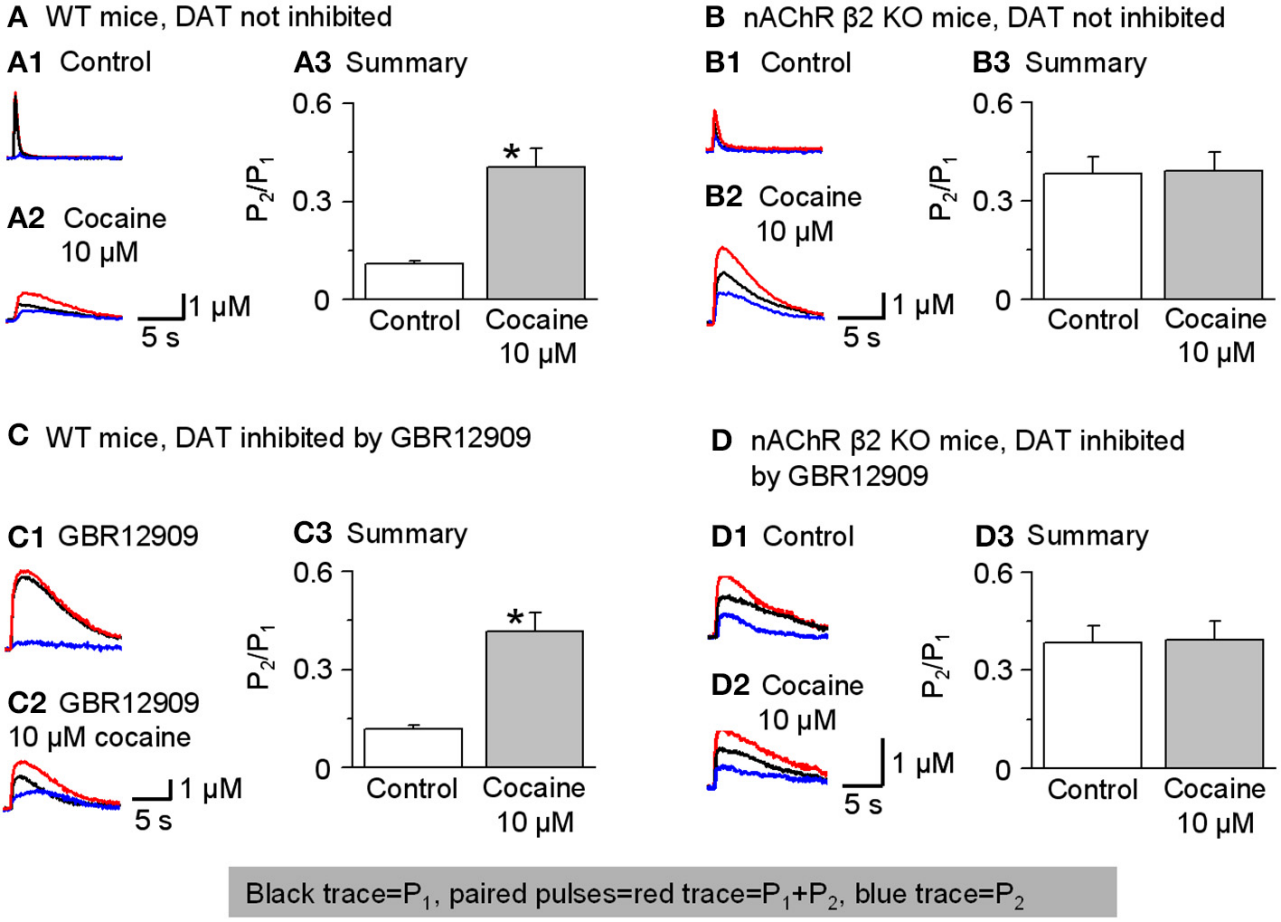
**Cocaine reduces paired-pulse depression of DA release in W-T but not in β 2-nAChR KO mice**. In this figure, P_1_ was the 1 stimulus pulse-evoked DA signal shown in black and P_2_ (shown in blue) was obtained by subtraction: the two paired pulses-evoked DA (red traces). **(A,A1–A3)** In W-T mice under control conditions (without inhibition of DATs), paired-pulse depression was strong, and the paired-pulse ratio (PPR, defined as P_2_/P_1_) was small. In 10 μM cocaine, the PPR significantly increased (*n* = 6). 1 μM sulpiride and SKF83566 were used in all these experiments to block local DA receptor interactions within the dorsolateral striatum. **(B,B1–B3)** In β 2-nAChR KO mice (without inhibition of DATs), 10 μM cocaine did not affect the PPR (*n* = 5). **(C,C1–C3)** In W-T mice with DAT inhibition by GBR12909 (5 μM) cocaine (10 μM) still enhances the PPR (*n* = 5). **(D,D1–D3)** In β 2-nAChR KO mice with inhibition of DATs, 10 μM cocaine did not affect the PPR (*n* = 5). ^*^p < 0.01.

To test this idea, we performed paired-pulse experiments using two stimuli separated by 50 ms (i.e., 20 Hz). The experiments were conducted in the presence of sulpiride (1 μM) to block autoinhibition by D_2_-like receptors and SKF83566 (1 μM) to block D_1_-like receptors on cholinergic interneurons to prevent changes in cholinergic tone in the striatum. The DA signal was quantified by the area under the curve because the 2 pulse protocol spreads the DA signal in time. The paired-pulse ratio (PPR) was defined as P_2_/P_1_. In the dorsolateral striatum of W-T mice, DA release displayed strong paired-pulse depression with a PPR of 0.12 ± 0.01, *n* = 7 (Figures 8A1,A3), consistent with published reports (Rice and Cragg, 2004; Zhang and Sulzer, 2004; Zhang et al., 2009a; Cachope et al., 2012; Threlfell et al., 2012). Although a high initial release probability is probably a key factor, the mechanism for this severe depletion of DA release is not established. In the presence of 10 μM cocaine, the PPR increased to 0.43 ± 0.06 (*n* = 5, *p* < 0.05; Figures 8A1–A3). Qualitatively similar results were obtained in the NAc shell: PPR was 0.47 ± 0.07, *n* = 5 in the control and 0.65 ± 0.08, *n* = 5 in 10 μM cocaine (not shown). These PPR numbers reflect the lower probability of release under control conditions in the NAc shell compared to the dorsal striatum, which was shown previously (Zhang et al., 2009a).

To examine further whether cocaine's mechanism of action on PPR was via nicotinic antagonism, we repeated the paired-pulse experiment in nAChR β 2 KO mice. In the absence of the β 2-containing nAChRs, the probability of release decreases especially for P_1_ (Zhou et al., 2001). Thus, in β 2 KO mice with no cocaine, the PPR was higher than in W-T mice, 0.38 ± 0.05, (*n* = 5, Figures 8B1,B3). More importantly, in the absence of functional β 2-containing nAChRs, 10 μM cocaine did not significantly affect the PPR consistent with cocaine mechanistically acting via nAChR inhibition: 0.39 ± 0.06 (*n* = 5, Figures 8B1–B3). Qualitatively similar results were obtained in the NAc shell: PPR was 0.68 ± 0.06 (*n* = 5) in the β 2 KO mice and 0.65 ± 0.06 (*n* = 5) when cocaine (10 μM) was applied (not shown). In the absence of β 2-containing nAChRs, cocaine did not influence the PPR.

To further demonstrate that cocaine is altering the PPR via nAChRs not owing to its inhibition of DATs, we repeated the experiments in the presence of GBR12909 (5 μM) to inhibit DATs as a control for cocaine's inhibition of DATs. Just as seen in Figures 8A,B, cocaine caused an increase in the PPR when nAChRs are present (Figures 8C1–C3) but not in β 2 KO mice (Figures 8D1–D3).

### Cocaine enhances phasic relative to tonic DA signals dependent on nAChRs

The earlier results showed that cocaine decreases DA release evoked by a 1 p stimulus. A low release probability can be overcome by the residual Ca^2+^ in axon terminals produced by high frequency stimulation (Abbott and Regehr, 2004). Therefore, we tested whether cocaine favors phasic DA release evoked by high frequency stimulation.

**Figure 9:**
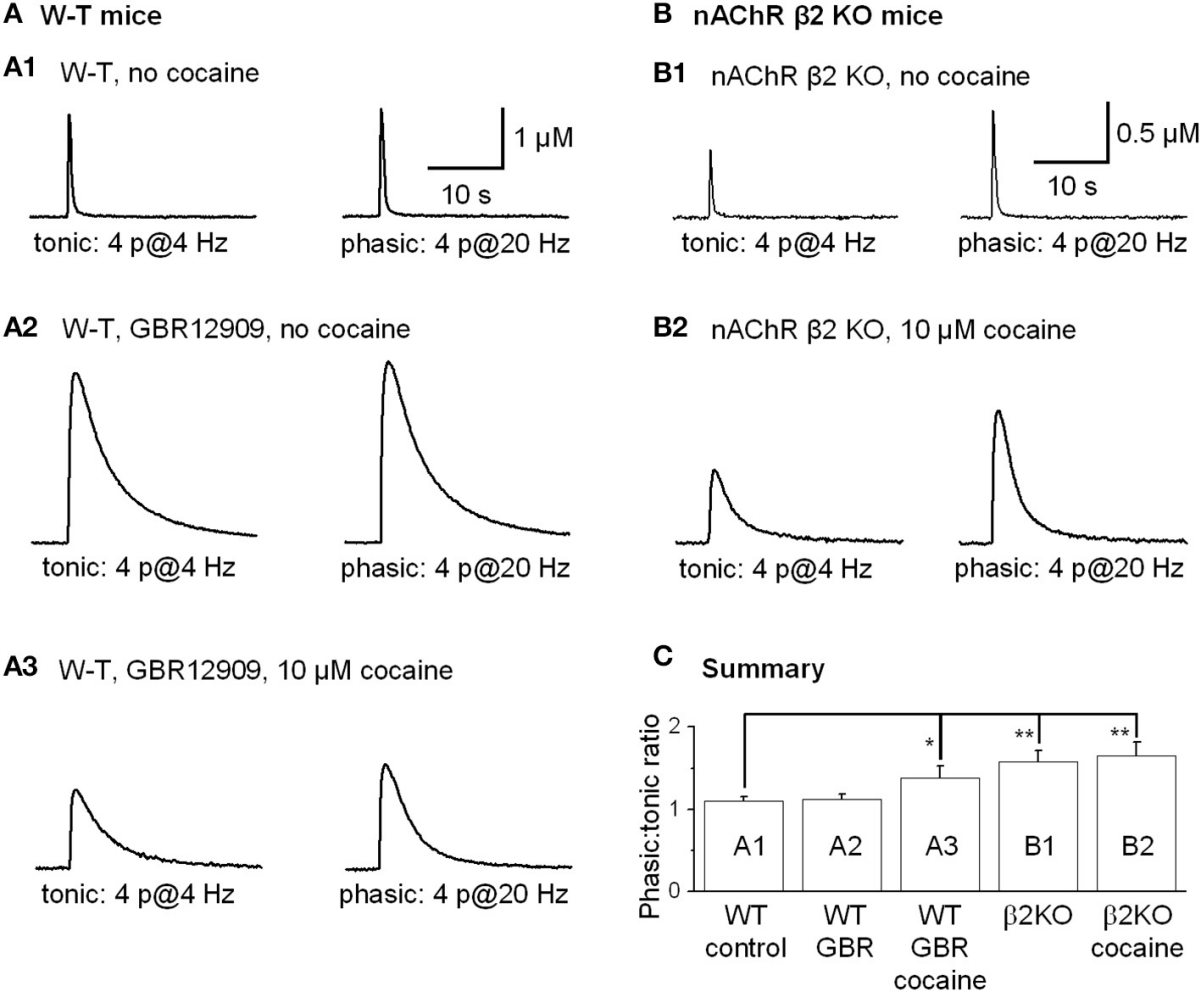
**Cocaine increased the phasic to tonic DA ratio in the dorsal striatum of W-T and nAChR KO mice**. Sulpiride and SKF83566 both at 1 μM were used to block DA receptor interactions within the striatum. **(A,A1)** The FCV measurements of DA signals evoked by lower frequency tonic stimulation (4 Hz, 4-pulses, left trace) and by phasic stimulation (20 Hz, 4-pulses, right trace) under control conditions had a phasic to tonic DA signal ratio close to 1. **(A,A2)** In 5 μM GBR12909 to inhibit DATs selectively (no cocaine), the DA signals evoked by tonic stimulation (left trace) and by phasic stimulation (right trace) showed a phasic to tonic ratio near 1. GBR12909 prolonged the DA signal duration but did not enhance the phasic to tonic DA ratio. **(A,A3)** In 10 μM cocaine, the DA signals evoked by tonic stimulation (left trace) and phasic stimulation (right trace) showed a larger phasic to tonic ratio. The DA signals were broader because cocaine inhibited DATs and, thus, prolonged the DA signal duration. **(B,B1)** Cocaine did not increase the phasic to tonic DA ratio in the dorsal striatum in β 2-nAChR KO Mice. The DA signal evoked by a tonic stimulation (4 Hz, 4-pulses, left trace) and phasic stimulation (20 Hz, 4-pulses, right trace) under control conditions. In β 2-nAChR KO mice the phasic to tonic DA signal ratio was larger than 1 and larger than in W-T mice. **(B,B2)** In 10 μM cocaine, the DA signals evoked by a tonic stimulation (left trace) and by a phasic stimulation (right trace) were prolonged, but the ratio of phasic to tonic signal was unchanged compared to the no cocaine condition. Cocaine did not dose-dependently enhance the phasic to tonic DA ratio in β 2-nAChR KO mice. Sulpiride and SKF83566 both at 1 μM were used to block DA receptor interactions within the striatum. **(C)** Summary graph showing the PPR under different conditions. ^*^p < 0.05, ^**^p < 0.01.

Tonic DA release was evoked by 4 stimulation pulses given at 4 Hz, and phasic DA release was mimicked by 4 pulses given at 20 Hz because rodent DA neurons fire bursting spikes with an intraburst frequency of ~20 Hz and with 3–5 spikes per burst, and the averaged DA neuron firing rate is about 4 Hz (Hyland et al., 2002; Schultz, 2002; Zhang et al., 2009b). Under control conditions in the dorsolateral striatum, phasic and tonic DA release was very similar (Rice and Cragg, 2004; Zhang and Sulzer, 2004; Zhang et al., 2009a), with the ratio of phasic to tonic DA release being 1.10 ± 0.05, *n* = 10 (Figures 9A1,C). Inhibition of DATs by GBR12909 (up to 5 μM) did not affect the phasic to tonic ratio (Figures 9A2,C). In contrast, 10 μM cocaine reduced the tonic DA release more strongly than the phasic DA signal increasing the phasic to tonic DA ratio to 1.38 ± 0.15 (*n* = 8, *p* < 0.05, Figures 9A3,C). To test whether cocaine was acting via inhibition of nAChRs to influence the phasic to tonic ratio, we repeated the experiment in nAChR β 2 KO mice. Under control conditions in β 2 KO mice, the phasic to tonic DA release ratio was 1.58 ± 0.14 (*n* = 6, Figures 9B1,C), which is higher than in W-T mice. Equally important, cocaine (up to 10 μM) did not alter the phasic to tonic DA ratio in β 2 KO mice, 1.65 ± 0.17 (*n* = 6, Figures 9B2,C). These data support the hypothesis that cocaine acts via inhibition of nAChRs to increase the phasic to tonic DA ratio.

## Discussion

The results from our present study indicate that at concentrations achieved by cocaine abusers, cocaine inhibits nAChRs and increases the ratio of phasic to tonic DA release. For example, cocaine (10 μM) decreased DA release evoked by a single, isolated stimulation (1 p) by ~70%, but that DA signaling loss was partially recovered during a stimulus train (4 p at 20 Hz). Because this relative enhancement of phasic signaling is occurring while cocaine also inhibits DA reuptake (i.e., inhibits DATs), the phasic DA signals are larger during cocaine abuse. Salient, reward-related DA signals arising from phasic DA neuron firing (Grace, 2000) will be highly exaggerated in cocaine concentrations greater than about 2 μM, which are easily achieved by cocaine abusers (Mittleman and Wetli, 1984; Evans et al., 1996; Ward et al., 1997; Fowler et al., 1998).

It must be kept in mind, however, that the striatal brain slice preparation and the exogenous electrical stimulation of the striatal tissue to evoke DA release is a reduced experimental model system that allows us to examine the different aspects of cocaine's effect on the DA signal in the striatum. As seen in Figure 6B, under these experimental conditions, cocaine's influence acting exclusively via nAChRs is mainly to diminish the DA release that would be even larger owing to cocaine's inhibition of DATs. By inhibiting nAChRs, the stimulus evoked DA release is not as large as would be achieved if the nAChRs were not partially inhibited by cocaine (Zhang et al., 2009a). In intact animals, cocaine's effects are more complex. For example, cocaine's inhibition of DA uptake increases the basal extracellular DA level that may activate the inhibitory D2 autoreceptors that in turn reduce DA release (Schmitz et al., 2002), complicating the determination of the physiological functions of the individual receptors and neurotransmitter systems.

### Nicotinic antagonism by cocaine regulates DA release in the striatum

The results from this study indicate three different cocaine concentration ranges that induce different antagonist actions (best seen in Figure 6). At low concentrations, below 1 μM (IC_50_ ≅ 0.5 μM) (Ritz et al., 1987), cocaine inhibits DATs and elevates extracellular DA by reducing DA reuptake. This is probably cocaine's most common effect in cocaine abusers for the simple reason that low cocaine concentrations are more common and last longer than the high cocaine peak (Evans et al., 1996; Ward et al., 1997; Fowler et al., 1998). At intermediate concentrations above 2 μM (IC_50_ of 4.3 μM), also commonly achieved by cocaine abusers, cocaine begins to inhibit nAChRs (Figure 3B) and, thereby, alters DA signaling via nicotinic mechanisms (Zhou et al., 2001; Rice and Cragg, 2004; Zhang and Sulzer, 2004). At high concentrations above 10 μM (IC_50_ ≅ 26.4 μM) that can be reached during a large cocaine dosing, local anesthetic-like effects begin (Mittleman and Wetli, 1984; O'Leary and Chahine, 2002). The mechanism for cocaine's stronger inhibition of DA release than that of voltage-gated Na current see in our data is not known; we speculate that the Na current at the DA axon fibers and terminals may be more sensitive to cocaine; the extensive DA axon fiber bifurcations may also increase the sensitivity of the action potential generation and propagation to cocaine inhibition (Matsuda et al., 2009; Debanne et al., 2011).

Our results show that in striatal slices from W-T mice, cocaine inhibited β 2-containing nAChR-dependent DA release (from 1 p stimulation) with an IC_50_ of 4.3 μM (Figure 3B). In contrast, a six time greater cocaine concentration is needed to depress 1 p-stimulation DA release from β 2-containing nAChR KO mice with an IC_50_ of 26.4 μM (Figure 5B). The data indicate that cocaine's inhibition of DA release is mediated by β 2 nAChRs on DA fibers and terminals that normally regulate DA release (Zhou et al., 2001; Grady et al., 2002; Rice and Cragg, 2004; Salminen et al., 2004; Zhang and Sulzer, 2004; Zhang et al., 2009a). This conclusion is also supported by our finding that in DA neuron somata, cocaine inhibited the β 2^*^ nAChR current (Figure 2B), consistent with previous studies of cloned α4β 2 nAChRs in expression systems (Damaj et al., 1999; Francis et al., 2000). The inhibition of nAChR currents and DA release was quantitatively mimicked by the selective nAChR antagonist DHβ E (Figure 2B2). Because cocaine often reaches greater than 2 μM in the brains of abusers (Mittleman and Wetli, 1984; Evans et al., 1996; Ward et al., 1997; Fowler et al., 1998), our results indicate that biologically relevant cocaine levels directly alter DA release.

The observation that weak cocaine inhibition of nAChRs (Figure 2B) leads to a greatly amplified inhibition of DA release (Figure 3A) likely arises from the site of nAChR action. nAChRs on DA fibers and terminals regulate action potential propagation and presynaptic calcium signals. For example, nAChRs mediate direct and indirect Ca^2+^ elevation within axon terminals (Vernino et al., 1992, 1994; Lena et al., 1993; McGehee et al., 1995; Gray et al., 1996; Rathouz et al., 1996). Neurotransmitter release is related to a high power (≅4th) of intra-terminal Ca^2+^ (Zucker and Regehr, 2002; Lou et al., 2005). Consequently, even a small decrease in the nAChR-initiated presynaptic Ca^2+^ signal or depolarization would induce a larger decrease in DA release. This conclusion is supported by the quantitatively similar data obtained with the specific nAChR inhibitor, DHβ E, which decreases nAChR-dependent DA release more strongly than nAChR-mediated currents (Figures 2B, 3A).

A number of recent studies have shown that nAChR activity on DA fibers and terminals regulates the relationship between afferent action potentials and DA release. The nAChRs on DA fibers and terminals increase the initial DA release probability, enhancing tonic DA signals (Grady et al., 2002; Rice and Cragg, 2004; Salminen et al., 2004; Zhang and Sulzer, 2004; Exley and Cragg, 2008; Zhang et al., 2009a). nAChR activation may also directly evoke action potentials in DA axon terminals and thus DA release (Cachope et al., 2012; Threlfell et al., 2012). Inhibiting or desensitizing striatal nAChRs decreases tonic DA release, but phasic DA release arising from stimulus trains is not inhibited. This nicotinic effect increases the ratio of phasic to tonic DA release arising from the biologically complex series of action potentials along DA fibers (Rice and Cragg, 2004; Zhang and Sulzer, 2004; Zhang et al., 2009a). By inhibiting nAChRs, cocaine influences this nicotinic mechanism that alters the frequency dependence of DA release favoring phasic signals.

### Biological implications

**Figure 10:**
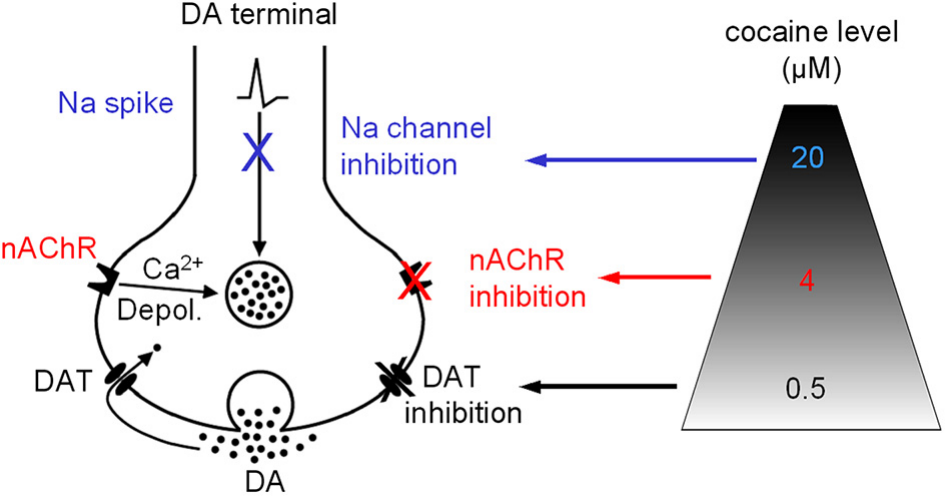
**Diagram showing that cocaine may inhibit DAT-mediated DA uptake and DA release via nAChRs and Na channels in a dose-dependent manner**. Depol., depolarization.

Our data indicate that besides inhibiting DAT, the most common effect on the DA system, cocaine, at concentrations (around 4 μM) readily achievable in cocaine abusers (Evans et al., 1996; Ward et al., 1997; Fowler et al., 1998), may also, via inhibiting nAChRs, alter DA release property. On rare occasions when the abuser uses very high doses of cocaine leading to a cocaine level ≥20 μM, cocaine's anesthetic effect may be triggered (Figure 10). The functional importance of our data obtained in brain slices is reflected in several behavioral studies showing that nicotinic agonism increases the abusive potential of cocaine whereas inhibition of nAChRs decreases cocaine reinforced behaviors (Reid et al., 1998; Zachariou et al., 2001; Schoffelmeer et al., 2002; Blokhina et al., 2005; Champtiaux et al., 2006; Zanetti et al., 2006). Particularly, pharmacological or genetic inactivation of nAChRs before exposure to cocaine was reported to disrupt place preference to cocaine, whereas low doses of nicotine were able to lower the threshold for cocaine induced place preference, and nAChR β 2 KO mice showed decreased cocaine induced place preference (Zachariou et al., 2001). It was also reported that nAChR inhibition by mecamylamine dose dependently suppressed cocaine self-administration (Blokhina et al., 2005). These literature data clearly indicate that nAChRs are involved in cocaine's addictive processes, although the underlying neural mechanisms are likely to be complex. Therefore, it is reasonable to conclude that the mechanistic effects reported in this study contribute to those *in vivo* findings: acting via inhibition of nAChRs, cocaine increases the ratio of phasic to tonic DA release and thus potentially enhances its reinforcing abilities (Goto and Grace, 2005).

### Conflict of interest statement

The authors declare that the research was conducted in the absence of any commercial or financial relationships that could be construed as a potential conflict of interest.

## References

[B1] Abbott L. F. Regehr W. G. (2004). Synaptic computation. Nature 431, 796–803 10.1038/nature03010 15483601

[B2] Alkondon M. Albuquerque E. X. (1993). Diversity of nicotinic acetylcholine receptors in rat hippocampal neurons. I. Pharmacological and functional evidence for distinct structural subtypes. J. Pharmacol. Exp. Ther. 265, 1455–1473 8510022

[B3] Beckstead M. J. Williams J. T. (2007). Long-term depression of a dopamine IPSC. J. Neurosci. 27, 2074–2080 10.1523/JNEUROSCI.3251-06.2007 17314302 PMC6673562

[B4] Blokhina E. A. Kashkin V. A. Zvartau E. E. Danysz W. Bespalov A. Y. (2005). Effects of nicotinic and NMDA receptor channel blockers on intravenous cocaine and nicotine self-administration in mice. Eur. Neuropsychopharmacol. 15, 219–225 10.1016/j.euroneuro.2004.07.005 15695068

[B5] Bonci A. Bernardi G. Grillner P. Mercuri N. B. (2003). The dopamine-containing neuron: maestro or simple musician in the orchestra of addiction? Trends Pharmacol. Sci. 24, 172–177 10.1016/S0165-6147(03)00068-3 12707003

[B6] Cachope R. Mateo Y. Mathur B. N. Irving J. Wang H. L. Morales M. (2012). Selective activation of cholinergic interneurons enhances accumbal phasic dopamine release: setting the tone for reward processing. Cell Rep. 2, 33–41 10.1016/j.celrep.2012.05.011 22840394 PMC3408582

[B7] Champtiaux N. Gotti C. Cordero-Erausquin M. David D. J. Przybylski C. Lena C. (2003). Subunit composition of functional nicotinic receptors in dopaminergic neurons investigated with knock-out mice. J. Neurosci. 23, 7820–7829 12944511 10.1523/JNEUROSCI.23-21-07820.2003 PMC6740613

[B8] Champtiaux N. Kalivas P. W. Bardo M. T. (2006). Contribution of dihydro-beta-erythroidine sensitive nicotinic acetylcholine receptors in the ventral tegmental area to cocaine-induced behavioral sensitization in rats. Behav. Brain Res. 168, 120–126 10.1016/j.bbr.2005.10.017 16313978

[B9] Chen R. Tilley M. R. Wei H. Zhou F. Zhou F. M. Ching S. (2006). Abolished cocaine reward in mice with a cocaine-insensitive dopamine transporter. Proc. Natl. Acad. Sci. U.S.A. 103, 9333–9338 10.1073/pnas.0600905103 16754872 PMC1482610

[B10] Damaj M. I. Slemmer J. E. Carroll F. I. Martin B. R. (1999). Pharmacological characterization of nicotine's interaction with cocaine and cocaine analogs. J. Pharmacol. Exp. Ther. 289, 1229–1236 10336510

[B11] Debanne D. Campanac E. Bialowas A. Carlier E. Alcaraz G. (2011). Axon physiology. Physiol. Rev. 91, 555–602 10.1152/physrev.00048.2009 21527732

[B12] Ding S. Wei W. Zhou F. M. (2011). Molecular and functional differences in voltage-activated sodium currents between GABA projection neurons and dopamine neurons in the substantia nigra. J. Neurophysiol. 106, 3019–3034 10.1152/jn.00305.2011 21880943 PMC3234097

[B13] Drenan R. M. Grady S. R. Steele A. D. McKinney S. Patzlaff N. E. McIntosh J. M. (2010). Cholinergic modulation of locomotion and striatal dopamine release is mediated by alpha6alpha4^*^ nicotinic acetylcholine receptors. J. Neurosci. 30, 9877–9889 10.1523/JNEUROSCI.2056-10.2010 20660270 PMC3390922

[B14] Evans S. M. Cone E. J. Henningfield J. E. (1996). Arterial and venous cocaine plasma concentrations in humans: relationship to route of administration, cardiovascular effects and subjective effects. J. Pharmacol. Exp. Ther. 279, 1345–1356 8968359

[B15] Exley R. Cragg S. J. (2008). Presynaptic nicotinic receptors: a dynamic and diverse cholinergic filter of striatal dopamine neurotransmission. Br. J. Pharmacol. 153(Suppl. 1), S283–S297 10.1038/sj.bjp.0707510 18037926 PMC2268048

[B16] Exley R. Maubourguet N. David V. Eddine R. Evrard A. Pons S. (2011). Distinct contributions of nicotinic acetylcholine receptor subunit {alpha}4 and subunit {alpha}6 to the reinforcing effects of nicotine. Proc. Natl. Acad. Sci. U.S.A. 108, 7577–7582 10.1073/pnas.1103000108 21502501 PMC3088627

[B17] Ford C. P. Mark G. P. Williams J. T. (2006). Properties and opioid inhibition of mesolimbic dopamine neurons vary according to target location. J. Neurosci. 26, 2788–2797 10.1523/JNEUROSCI.4331-05.2006 16525058 PMC3623681

[B18] Fowler J. S. Volkow N. D. Logan J. Gatley S. J. Pappas N. King P. (1998). Measuring dopamine transporter occupancy by cocaine *in vivo*: radiotracer considerations. Synapse 28, 111–116 10.1002/(SICI)1098-2396(199802)28:2<111::AID-SYN1>3.0.CO;2-E 9450511

[B19] Francis M. M. Vazquez R. W. Papke R. L. Oswald R. E. (2000). Subtype-selective inhibition of neuronal nicotinic acetylcholine receptors by cocaine is determined by the alpha4 and beta4 subunits. Mol. Pharmacol. 58, 109–119 10860932 10.1124/mol.58.1.109

[B20] Goto Y. Grace A. A. (2005). Dopaminergic modulation of limbic and cortical drive of nucleus accumbens in goal-directed behavior. Nat. Neurosci. 8, 805–812 10.1038/nn1471 15908948

[B21] Gould E. Woolf N. J. Butcher L. L. (1989). Cholinergic projections to the substantia nigra from the pedunculopontine and laterodorsal tegmental nuclei. Neuroscience 28, 611–623 2710334 10.1016/0306-4522(89)90008-0

[B22] Grace A. A. (2000). The tonic/phasic model of dopamine system regulation and its implications for understanding alcohol and psychostimulant craving. Addiction 95(Suppl. 2), S119–S128 10.1046/j.1360-0443.95.8s2.1.x 11002907

[B23] Grady S. R. Murphy K. L. Cao J. Marks M. J. McIntosh J. M. Collins A. C. (2002). Characterization of nicotinic agonist-induced [(3)H]dopamine release from synaptosomes prepared from four mouse brain regions. J. Pharmacol. Exp. Ther. 301, 651–660 10.1124/jpet.301.2.651 11961070

[B24] Gray R. Rajan A. S. Radcliffe K. A. Yakehiro M. Dani J. A. (1996). Hippocampal synaptic transmission enhanced by low concentrations of nicotine. Nature 383, 713–716 10.1038/383713a0 8878480

[B25] Hyland B. I. Reynolds J. N. Hay J. Perk C. G. Miller R. (2002). Firing modes of midbrain dopamine cells in the freely moving rat. Neuroscience 114, 475–492 10.1016/S0306-4522(02)00267-1 12204216

[B26] Hyman S. E. Malenka R. C. Nestler E. J. (2006). Neural mechanisms of addiction: the role of reward-related learning and memory. Annu. Rev. Neurosci. 29, 565–598 10.1146/annurev.neuro.29.051605.113009 16776597

[B27] Jones I. W. Bolam J. P. Wonnacott S. (2001). Presynaptic localisation of the nicotinic acetylcholine receptor beta2 subunit immunoreactivity in rat nigrostriatal dopaminergic neurones. J. Comp. Neurol. 439, 235–247 10.1002/cne.1345 11596051

[B28] Jones S. R. Garris P. A. Kilts C. D. Wightman R. M. (1995). Comparison of dopamine uptake in the basolateral amygdaloid nucleus, caudate-putamen, and nucleus accumbens of the rat. J. Neurochem. 64, 2581–2589 10.1046/j.1471-4159.1995.64062581.x 7760038

[B29] Lena C. Changeux J. P. Mulle C. (1993). Evidence for “preterminal” nicotinic receptors on GABAergic axons in the rat interpeduncular nucleus. J. Neurosci. 13, 2680–2688 8501532 10.1523/JNEUROSCI.13-06-02680.1993 PMC6576498

[B30] Li W. Doyon W. M. Dani J. A. (2011). Acute *in vivo* nicotine administration enhances synchrony among dopamine neurons. Biochem. Pharmacol. 82, 977–983 10.1016/j.bcp.2011.06.006 21684263 PMC3162092

[B31] Lou X. Scheuss V. Schneggenburger R. (2005). Allosteric modulation of the presynaptic Ca^2+^ sensor for vesicle fusion. Nature 435, 497–501 10.1038/nature03568 15917809

[B32] Mansvelder H. D. McGehee D. S. (2000). Long-term potentiation of excitatory inputs to brain reward areas by nicotine. Neuron 27, 349–357 10.1016/S0896-6273(00)00042-8 10985354

[B33] Matsuda W. Furuta T. Nakamura K. C. Hioki H. Fujiyama F. Arai R. (2009). Single nigrostriatal dopaminergic neurons form widely spread and highly dense axonal arborizations in the neostriatum. J. Neurosci. 29, 444–453 10.1523/JNEUROSCI.4029-08.2009 19144844 PMC6664950

[B34] McGehee D. S. Heath M. J. Gelber S. Devay P. Role L. W. (1995). Nicotine enhancement of fast excitatory synaptic transmission in CNS by presynaptic receptors. Science 269, 1692–1696 10.1126/science.7569895 7569895

[B35] Mittleman R. E. Wetli C. V. (1984). Death caused by recreational cocaine use. An update. JAMA 252, 1889–1893 6471319

[B36] Nelson A. B. Hammack N. Yang C. F. Shah N. M. Seal R. P. Kreitzer A. C. (2014). Striatal cholinergic interneurons drive GABA release from dopamine terminals. Neuron 82, 63–70 10.1016/j.neuron.2014.01.023 24613418 PMC3976769

[B37] Neuhoff H. Neu A. Liss B. Roeper J. (2002). I(h) channels contribute to the different functional properties of identified dopaminergic subpopulations in the midbrain. J. Neurosci. 22, 1290–1302 11850457 10.1523/JNEUROSCI.22-04-01290.2002 PMC6757558

[B38] Nicola S. M. Deadwyler S. A. (2000). Firing rate of nucleus accumbens neurons is dopamine-dependent and reflects the timing of cocaine-seeking behavior in rats on a progressive ratio schedule of reinforcement. J. Neurosci. 20, 5526–5537 10884336 10.1523/JNEUROSCI.20-14-05526.2000 PMC6772321

[B39] Oakman S. A. Faris P. L. Kerr P. E. Cozzari C. Hartman B. K. (1995). Distribution of pontomesencephalic cholinergic neurons projecting to substantia nigra differs significantly from those projecting to ventral tegmental area. J. Neurosci. 15, 5859–5869 7666171 10.1523/JNEUROSCI.15-09-05859.1995 PMC6577686

[B40] O'Leary M. E. Chahine M. (2002). Cocaine binds to a common site on open and inactivated human heart (Na(v)1.5) sodium channels. J. Physiol. (Lond.) 541, 701–716 10.1113/jphysiol.2001.016139 12068034 PMC2290378

[B41] Picciotto M. R. Zoli M. Lena C. Bessis A. Lallemand Y. Le Novere N. (1995). Abnormal avoidance learning in mice lacking functional high-affinity nicotine receptor in the brain. Nature 374, 65–67 10.1038/374065a0 7870173

[B42] Pidoplichko V. I. Dani J. A. (2005). Applying small quantities of multiple compounds to defined locations of *in vitro* brain slices. J. Neurosci. Methods 142, 55–66 10.1016/j.jneumeth.2004.07.012 15652617

[B43] Pidoplichko V. I. De Biasi M. Williams J. T. Dani J. A. (1997). Nicotine activates and desensitizes midbrain dopamine neurons. Nature 390, 401–404 10.1038/37120 9389479

[B44] Pristupa Z. B. Wilson J. M. Hoffman B. J. Kish S. J. Niznik H. B. (1994). Pharmacological heterogeneity of the cloned and native human dopamine transporter: disassociation of [3H]WIN 35,428 and [3H]GBR 12,935 binding. Mol. Pharmacol. 45, 125–135 8302271

[B45] Quik M. McIntosh J. M. (2006). Striatal alpha6^*^ nicotinic acetylcholine receptors: potential targets for parkinson's disease therapy. J. Pharmacol. Exp. Ther. 316, 481–489 10.1124/jpet.105.094375 16210393

[B46] Rathouz M. M. Vijayaraghavan S. Berg D. K. (1996). Elevation of intracellular calcium levels in neurons by nicotinic acetylcholine receptors. Mol. Neurobiol. 12, 117–131 10.1007/BF02740649 8818146

[B47] Reid M. S. Mickalian J. D. Delucchi K. L. Hall S. M. Berger S. P. (1998). An acute dose of nicotine enhances cue-induced cocaine craving. Drug Alcohol Depend. 49, 95–104 10.1016/S0376-8716(97)00144-0 9543646

[B48] Rice M. E. Cragg S. J. (2004). Nicotine amplifies reward-related dopamine signals in striatum. Nat. Neurosci. 7, 583–584 10.1038/nn1244 15146188

[B49] Ritz M. C. Lamb R. J. Goldberg S. R. Kuhar M. J. (1987). Cocaine receptors on dopamine transporters are related to self-administration of cocaine. Science 237, 1219–1223 10.1126/science.2820058 2820058

[B50] Salminen O. Murphy K. L. McIntosh J. M. Drago J. Marks M. J. Collins A. C. (2004). Subunit composition and pharmacology of two classes of striatal presynaptic nicotinic acetylcholine receptors mediating dopamine release in mice. Mol. Pharmacol. 65, 1526–1535 10.1124/mol.65.6.1526 15155845

[B51] Schmitz Y. Schmauss C. Sulzer D. (2002). Altered dopamine release and uptake kinetics in mice lacking D2 receptors. J. Neurosci. 22, 8002–8009 12223553 10.1523/JNEUROSCI.22-18-08002.2002 PMC6758092

[B52] Schoffelmeer A. N. De Vries T. J. Wardeh G. Van De Ven H. W. Vanderschuren L. J. (2002). Psychostimulant-induced behavioral sensitization depends on nicotinic receptor activation. J. Neurosci. 22, 3269–3276 11943828 10.1523/JNEUROSCI.22-08-03269.2002 PMC6757535

[B53] Schultz W. (2002). Getting formal with dopamine and reward. Neuron 36, 241–263 10.1016/S0896-6273(02)00967-4 12383780

[B54] Threlfell S. Lalic T. Platt N. J. Jennings K. A. Deisseroth K. Cragg S. J. (2012). Striatal dopamine release is triggered by synchronized activity in cholinergic interneurons. Neuron 75, 58–64 10.1016/j.neuron.2012.04.038 22794260

[B55] Vernino S. Amador M. Luetje C. W. Patrick J. Dani J. A. (1992). Calcium modulation and high calcium permeability of neuronal nicotinic acetylcholine receptors. Neuron 8, 127–134 10.1016/0896-6273(92)90114-S 1370370

[B56] Vernino S. Rogers M. Radcliffe K. A. Dani J. A. (1994). Quantitative measurement of calcium flux through muscle and neuronal nicotinic acetylcholine receptors. J. Neurosci. 14, 5514–5524 8083751 10.1523/JNEUROSCI.14-09-05514.1994 PMC6577067

[B57] Ward A. S. Haney M. Fischman M. W. Foltin R. W. (1997). Binge cocaine self-administration in humans: intravenous cocaine. Psychopharmacology (Berl.) 132, 375–381 10.1007/s002130050358 9298515

[B58] Wise R. A. (2004). Dopamine, learning and motivation. Nat. Rev. Neurosci. 5, 483–494 10.1038/nrn1406 15152198

[B59] Woolf N. J. Butcher L. L. (1981). Cholinergic neurons in the caudate-putamen complex proper are intrinsically organized: a combined Evans blue and acetylcholinesterase analysis. Brain Res. Bull. 7, 487–507 7317794 10.1016/0361-9230(81)90004-6

[B60] Woolf N. J. Butcher L. L. (1986). Cholinergic systems in the rat brain: III. Projections from the pontomesencephalic tegmentum to the thalamus, tectum, basal ganglia, and basal forebrain. Brain Res. Bull. 16, 603–637 3742247 10.1016/0361-9230(86)90134-6

[B61] Wooltorton J. R. Pidoplichko V. I. Broide R. S. Dani J. A. (2003). Differential desensitization and distribution of nicotinic acetylcholine receptor subtypes in midbrain dopamine areas. J. Neurosci. 23, 3176–3185 12716925 10.1523/JNEUROSCI.23-08-03176.2003 PMC6742341

[B62] Xu W. Orr-Urtreger A. Nigro F. Gelber S. Sutcliffe C. B. Armstrong D. (1999). Multiorgan autonomic dysfunction in mice lacking the beta2 and the beta4 subunits of neuronal nicotinic acetylcholine receptors. J. Neurosci. 19, 9298–9305 10531434 10.1523/JNEUROSCI.19-21-09298.1999 PMC6782888

[B63] Zachariou V. Caldarone B. J. Weathers-Lowin A. George T. P. Elsworth J. D. Roth R. H. (2001). Nicotine receptor inactivation decreases sensitivity to cocaine. Neuropsychopharmacology 24, 576–589 10.1016/S0893-133X(00)00224-4 11282258

[B64] Zanetti L. De Kerchove D'exaerde A. Zanardi A. Changeux J. P. Picciotto M. R. Zoli M. (2006). Inhibition of both alpha7^*^ and beta2^*^ nicotinic acetylcholine receptors is necessary to prevent development of sensitization to cocaine-elicited increases in extracellular dopamine levels in the ventral striatum. Psychopharmacology (Berl.) 187, 181–188 10.1007/s00213-006-0419-y 16826402

[B65] Zhang H. Sulzer D. (2004). Frequency-dependent modulation of dopamine release by nicotine. Nat. Neurosci. 7, 581–582 10.1038/nn1243 15146187

[B66] Zhang L. Doyon W. M. Clark J. J. Phillips P. E. Dani J. A. (2009a). Controls of tonic and phasic dopamine transmission in the dorsal and ventral striatum. Mol. Pharmacol. 76, 396–404 10.1124/mol.109.056317 19460877 PMC2713129

[B67] Zhang T. Zhang L. Liang Y. Siapas A. G. Zhou F. M. Dani J. A. (2009b). Dopamine signaling differences in the nucleus accumbens and dorsal striatum exploited by nicotine. J. Neurosci. 29, 4035–4043 10.1523/JNEUROSCI.0261-09.2009 19339599 PMC2743099

[B68] Zhang T. A. Placzek A. N. Dani J. A. (2010). *In vitro* identification and electrophysiological characterization of dopamine neurons in the ventral tegmental area. Neuropharmacology 59, 431–436 10.1016/j.neuropharm.2010.06.004 20600174 PMC2946471

[B69] Zhou F. M. Liang Y. Dani J. A. (2001). Endogenous nicotinic cholinergic activity regulates dopamine release in the striatum. Nat. Neurosci. 4, 1224–1229 10.1038/nn769 11713470

[B70] Zucker R. S. Regehr W. G. (2002). Short-term synaptic plasticity. Annu. Rev. Physiol. 64, 355–405 10.1146/annurev.physiol.64.092501.114547 11826273

